# Editorial Comment on Firth et al. (2019)

**DOI:** 10.1097/PSY.0000000000000805

**Published:** 2020-04-07

**Authors:** Willem J. Kop, Benjamin P. Chapman

**Affiliations:** From the Department of Medical and Clinical Psychology, Center of Research on Psychology in Somatic diseases (CoRPS), Tilburg University (Kop), Tilburg, the Netherlands; and Departments of Psychiatry and Public Health Sciences (Chapman), University of Rochester Medical Center, Rochester, New York.

**Keywords:** meta-analysis

## Abstract

This Editorial Comment addresses an article by Firth et al. published in the February issue of 2019.

The editorial team of *Psychosomatic Medicine* was asked to reevaluate content details of an article published in the February 2019 issue of the journal. Specifically, in this issue, Firth et al. (1) described a systematic review and meta-analysis of the effects of dietary interventions on symptoms of depression and anxiety, including data from 16 randomized controlled trials with a total of 45,826 participants. Potential issues were raised about three of the included trials (combined n = 446). To address these issues, the journal editorial team decided to publish this Editorial Comment outlining the potential issues raised (as detailed hereinafter), contacted the authors (i.e., Firth and colleagues) with a request to respond to these issues and to provide alternative point estimates from additional analyses, excluding the articles that raised concern. The detailed response by Firth and colleagues follows this Editorial Comment in this issue of the journal.

The specific comments were as follows:

1. It was commented that the article by Wardle et al. (2) was interpreted as revealing a statistically significant effect size of 1.7 favoring the dietary condition. However, the pooled effect estimates across the two measures of depressive symptoms used by Wardle et al. actually produce a substantially lower effect size (between 0.20 and 0.40; approximately 0.26) favoring the dietary condition.
2. The second comment disputed the inclusion of the article by Endevelt et al. (3), which randomly assigned 68 older adults to: “an intensive nutritional intervention led by a dietitian (DIT = dietetic Intervention treatment) or a control treatment group (MT = medical treatment) led by a physician.” Here, it was raised that because the participants in the MT control condition were provided with an educational booklet that “included information regarding the nutritional needs of older adults,” this control group could be perceived as inconsistent with the wording of the inclusion criteria by Firth et al., stating “Eligible studies were randomized controlled trials (RCTs) comparing the effect of dietary interventions to non-dietary control conditions.” It was therefore suggested that this article should be excluded in alternative analyses of the results.
3. The third issue concerned the inclusion of the article by Scheier et al. (4), which compared an educational intervention to a nutritional intervention for young women completing treatment for early-stage breast cancer; a better rationale for this inclusion was requested. Specifically, in summarizing their dietary intervention, Scheier et al. note “Each session provided information and encouragement on setting and attaining measurable goals for healthy eating and on the benefits of thinking positively about dealing adaptively with problems in life and living a healthy lifestyle.” Thus, because participants within the dietary intervention also engaged in discussions about the benefits of thinking positively about dealing adaptively with problems, it could be argued that this article should not have been included in the meta-analysis. Although the nondietary additional components were minimal, it is possible that the active dietary and control conditions could be influenced by these other nondietary aspects of the study.

We asked Firth and colleagues to respond to these issues and also to reanalyze the data after excluding the contested studies. We appreciate the efforts by Firth et al. to comply with this request (please see this issue of the journal for their detailed response).

In evaluating the concerns raised and the response by Firth et al., we concur that the effect size used for the study by Wardle et al. should have been lower than reported in the original article (1) (i.e., between 0.20 and 0.40 and not *g* = 1.7). For this reason, we are publishing an erratum in this issue. However, this difference does not affect the overall findings and interpretation of the original meta-analysis. We agree with Firth et al. that all 16 studies included in the study are consistent with the inclusion and exclusion criteria of the original meta-analysis. In the following paragraphs, we provide a perspective on the statistical issues involved in our evaluation of this reanalysis of the data of the original meta-analysis.

The questions point to two statistical principles that are useful to consider. First, in any reanalysis, revised point estimates must be considered within the variance estimates of the original effect size estimates. Standard errors (or confidence intervals derived from them) provide some perspective on the degree of uncertainty around the original estimated effect size. A “quick and dirty” check is whether the revised estimate lies outside the 95% confidence interval of the original estimate (formal tests of differences between parameter estimates are possible in some instances [5]). In random effects meta-analysis, error can arise from sampling error within or between studies. Including two studies from a different population would represent sampling error at the between-study level and if the original estimator has done its job, this source of error will be appropriately incorporated into standard error estimates of the meta-analytic effect estimate. This is an important reminder of the necessity to think in terms of the interval within which a “true” effect is likely to reside, rather than to reify a single point estimate. In their response, Firth et al. provide information that the point estimates based on the reanalysis of the data fall within the confidence intervals of the original report.

Second, the type of inverse-variance weighted pooling in meta-analysis links the impact of errors to the size of the study. If a data entry error leads to an effect size too large or too small, its impact on the overall results will be proportional to the weight exerted by the study (i.e., its sample size or estimation precision). Statistically, this tamps down the “noise” of small studies, a within-study source of error. One might classify a mis-keyed number in the data set as a sort of measurement error, and one for which there is no statistical fix unless it is detected. Correcting such an error and repeating the analysis represent the best possible solution; this has been done in the response by Firth and colleagues in this issue of the journal.

We appreciate having received the comments on the original meta-analysis published in *Psychosomatic Medicine* (1) as well as the authors’ detailed response to these comments, which are published in this issue of the journal. We consider the main findings of the original article sound and conclude that the results are robust even when taking into account the suggestions for alternative approaches to the meta-analysis. It is important that critical issues are brought to our attention, and we encourage our readership to continue doing so, as such comments help to improve the science published in our field.

Willem J. Kop, PhD, Editor-in-Chief

Benjamin Chapman, PhD, MPH, MS, Statistical Editor
